# Retraction: Nicotinamide Inhibits Alkylating Agent-Induced Apoptotic Neurodegeneration in the Developing Rat Brain

**DOI:** 10.1371/journal.pone.0301150

**Published:** 2024-03-21

**Authors:** The PLOS ONE Editors

Following the publication of this article [1], concerns were raised regarding multiple figures, including,

- Similarities were noted between the following:
  ○ Lanes 2–4 of the thalamus Bcl-2 panel in Figure 2B of this article [1] and lanes 1–3 of the hippocampus Bcl-2 panel in Fig 1B of an earlier article by the same author group [2].
  ○ The cortex Bcl-2 panel in Figure 2B of [1] appears similar to the thalamus Bcl-2 panel in Fig 1B of [2].
  ○ The thalamus cytosolic cytochrome-C panel in Figure 3 of [1] and the cortex cyto-C panel in Fig 2 of [2]
  ○ The β-actin panel in Figure 3 of [1] and the β-actin panel in Fig 5 of [2].
  ○ The β-actin panel in Figure 4B of [1] and the β-actin panel in Fig 1A of [2].
  ○ The panels in Figure 5H of [1] and Fig 7B of [2].
  ○ The top right of the panel Figure 5G of [1] and the bottom left of the panel in Fig 7E of [2].
  ○ The bottom of the panel in Figure 5I and top of the panel in Figure 5K.
- All western blot experiments present samples from two tissues with one loading control.

The first author (NU) provided western blot images described as historical data underlying some of the published panels. However, these do not present the full blot area, and no images were provided for loading control panels.

The first author acknowledged that the following panels in this article [1] are incorrect: all panels in Figure 2B; thalamus cytosolic cytochrome-C and β-actin panels in Figure 3; β-actin panels in Figures 4A and 4B; and Figures 5H and K. They provided alternative versions of these panels; however, in the absence of the original data for these figures, the concerns cannot be resolved.

The first author stated that β-actin loading control was analyzed for samples from cortex and thalamus, except in cases where no significant changes in β-actin expression were detected. In the absence of confirmation that a loading control was measured for every sample, the editors remain concerned regarding the reliability of all western blot experiments.

In light of the concerns affecting multiple figure panels that question the reliability of these data, the *PLOS ONE* Editors retract this article.

NU did not agree with the retraction. HYL, MIN, IU, JWS, and MOK either did not respond directly or could not be reached.

Figures 2B, 3, 4B, 5G, and 5H appear to report material from [2], published in July 2011 by Elsevier, which are not offered under a CC-BY license and are therefore excluded from this article’s [1] license. At the time of retraction, the article [1] was republished to note this exclusion in the legend for Figures 2–5 and the article’s copyright statement.

## References

[1] Ullah N, Lee HY, Naseer MI, Ullah I, Suh JW, Kim MO (2011) Nicotinamide Inhibits Alkylating Agent-Induced Apoptotic Neurodegeneration in the Developing Rat Brain. PLoS ONE 6(12): e27093. doi: 10.1371/journal.pone.0027093 22164206 PMC3229474

[2] Ullah N, Naseer MI, Ullah I, Lee HY, Koh PO, Kim MO (2011) Protective effect of pyruvate against ethanol-induced apoptotic neurodegenaration in the developing rat brain. Neuropharmacology 61:1248–1255. 10.1016/j.neuropharm.2011.06.031 21803053

